# Comparative analysis of RADAR vs. conventional techniques for AVF maturation in patients with blood viscosity and vessel elasticity-related diseases through fluid-structure interaction modeling: Anemia, hypertension, and diabetes

**DOI:** 10.1371/journal.pone.0296631

**Published:** 2024-01-16

**Authors:** Patcharaporn Wongchadakul, Suphalerk Lohasammakul, Phadungsak Rattanadecho

**Affiliations:** 1 Princess Srisavangavadhana College of Medicine, Chulabhorn Royal Academy, Bangkok, Thailand; 2 Department of Anatomy, Faculty of Medicine, Siriraj Hospital, Mahidol University, Bangkok, Thailand; 3 Center of Excellence in Electromagnetic Energy Utilization in Engineering (C.E.E.E.), Department of Mechanical Engineering, Faculty of Engineering, Thammasat University (Rangsit Campus), Pathumthani, Thailand; Coventry University, UNITED KINGDOM

## Abstract

**Purpose:**

This study aims to compare two surgical techniques, the standard Vein-to-Artery and the newer Artery-to-Vein (Radial Artery Deviation And Reimplantation; RADAR), for enhancing the success of Arterio-Venous Fistula maturation in end-stage renal disease patients. The impact of diseases like anemia, diabetes, hypertension, and chronic kidney disease were considered. The goals are to advance Arterio-Venous Fistula (AVF) surgery, improve patient outcomes, and contribute to evidence-based surgical guidelines.

**Methods:**

Fluid-structure interaction modeling was employed to investigate how hemodynamic and mechanical stresses impact arteriovenous fistula maturation, with a particular focus on the role of wall shear stress in determining maturation outcomes. The critical threshold for vessel injury was identified as wall shear stress values exceeding 35 N/m^2^, while stenosis formation was projected to occur at levels below 1 N/m^2^. This work introduced a novel approach by considering disease-related factors, including blood viscosity (anemia), and vessel elasticity (diabetes, hypertension, and chronic kidney diseases), which directly influence hemodynamics and the generation of wall shear stress. Furthermore, the model was designed to incorporate varying thicknesses and elasticities for both the vein and artery, accurately representing authentic vascular anatomy.

**Results:**

The RADAR technique has demonstrated superior performance compared to the standard technique by providing appropriate wall shear stress in critical regions and minimizing the risk of wall damage. Its use of a thicker vessel also reduces the risk of vessel injury, making it particularly effective for patients with Chronic Kidney Disease (CKD), hypertension, anemia, and diabetes, ensuring optimal blood flow and fewer complications. However, there are minor concerns about stenosis formation in hypertension and anemia cases, which could be mitigated by adjusting the anastomosis angle to be lower than 30°.

**Conclusion:**

Diabetes and hypertension have significant physiological effects that increase the risks associated with arteriovenous fistula maturation. The anemic condition resulting from CKD may help reduce vessel injury but raises concerns about potential stenosis formation. Despite these co-morbidities, the RADAR technique has demonstrated its ability to induce more favorable hemodynamic changes, promoting arteriovenous fistula maturation.

## Introduction

Hemodialysis is one of the mandatory treatments in end stage renal disease (ERSD). This procedure requires creating a vascular access and surgical creation of Arterio-Venous Fistula (AVF) is a common option [1]. This operation demands a post-operative period for fistula maturation before becoming a valid route for vascular access. Overall, the success rate of AVF maturation is approximately 50–65% [2, 3]. Therefore, failure of maturation is not uncommon and this complication usually results in the negative outcomes that lead morbidity and mortality [4]. There are various causes, including site of the AVF [5]. The most common site of failure is at the radial artery and cephalic vein [1, 4]. Regardless the site of AVF, the common mechanism is related to a response to the increased hemodynamic force of both the feeding artery and draining vein that leads to stenosis obstructing the fistula’s pathway [6, 7].

Hemodynamic and mechanical stresses are critical factors affecting AVF maturation. The former creates a turbulent flow that results in insufficient Wall Shear Stress (WSS), leading to stenosis. The latter occurs because of excessive pressure and consequently causes thickening of the vessel. The combination of both increases the risk of vessel stenosis and failure of fistula maturation [8]. Thus, a thorough understanding of the interaction between hemodynamics and mechanical structure, particularly WSS, is crucial to enhance outcomes for surgical creation of AVF.

The creation of an AVF can be achieved using either standard end vein-to-side artery anastomosis or a later approach of end artery-to-side vein anastomosis, known as Radial Artery Deviation And Reimplantation (RADAR) [9]. On one hand, the RADAR technique has been reported to improve rate of maturation and stenosis in comparison to that of the former [9], since it establishes a smoother flow pattern and as a result reduces the associated risks of high flow rates [10]. On the other hand, it requires sacrifice of the flow of radial artery distal to the AVF. Selection of these techniques depends on various factors, including patient characteristics, surgeon expertise, and individual considerations such as the presence of existing diseases that can influence hemodynamics.

Basically, some co-morbidities can influence blood vessel elasticity and subsequently affect AVF outcomes. These includes poor elasticity in diabetes (stiffer artery [11] and vein [12–14]) and hemodynamics and WSS changes in hypertension [15] that can lead to maturation failure [16–19]. For Chronic Kidney Disease (CKD), it can affect arterial elasticity per se [11] and it often associated with diabetes, a primary cause of kidney failure in 60% of dialysis patients [20, 21]. Understanding the effects of these diseases on AVF is therefore crucial. In addition, anemia is another physiologic consequence of CKD [22] that worsened along the disease progression. The decreased red blood cells (RBCs) volume and concentration influence blood viscosity [23, 24] that is a critical factor in WSS generation affecting AVF maturation [25–28].

A careful evaluation of these related factors is necessary when considering the optimal technique for AVF creation. Thus, this study takes the effects of vessel elasticity resulted from CKD, diabetes, and/or hypertension, as well as the shear-dependent viscosity associated with anemia into account for investigating their consequences in AVF maturation and proposing a numerical comparison of the two techniques by considering non-Newtonian blood flow and mechanical deformation of vessels. To analyze incompressible non-Newtonian blood flow within deformed vessels, a fluid-structure interaction model [29–31] employing the Navier-Stokes equations with a turbulent model is utilized. This advanced modeling approach incorporates different thicknesses and elasticities between the vein and artery, closely mimicking actual vascular anatomy and properties compared to previous works. This advanced modeling approach has demonstrated success in predicting AVF behavior, preventing maturation failure [32], and contributing to a better understanding of vascular biology, ultimately leading to improved solutions for AVF creation [33].

The objectives of this study are as follows: (1) to enhance our understanding of AVF surgery and develop a surgical approach that minimizes the occurrence of fistula immaturity, (2) to incorporate the blood properties specific to CKD patients to obtain more realistic results, (3) to provide surgical recommendations for patients with diabetes, hypertension, and anemia based on hemodynamics and mechanical engineering principles, and (4) to compare the efficacy of different surgical techniques from an engineering standpoint.

## Formulation of the problem

**Fig 1:**
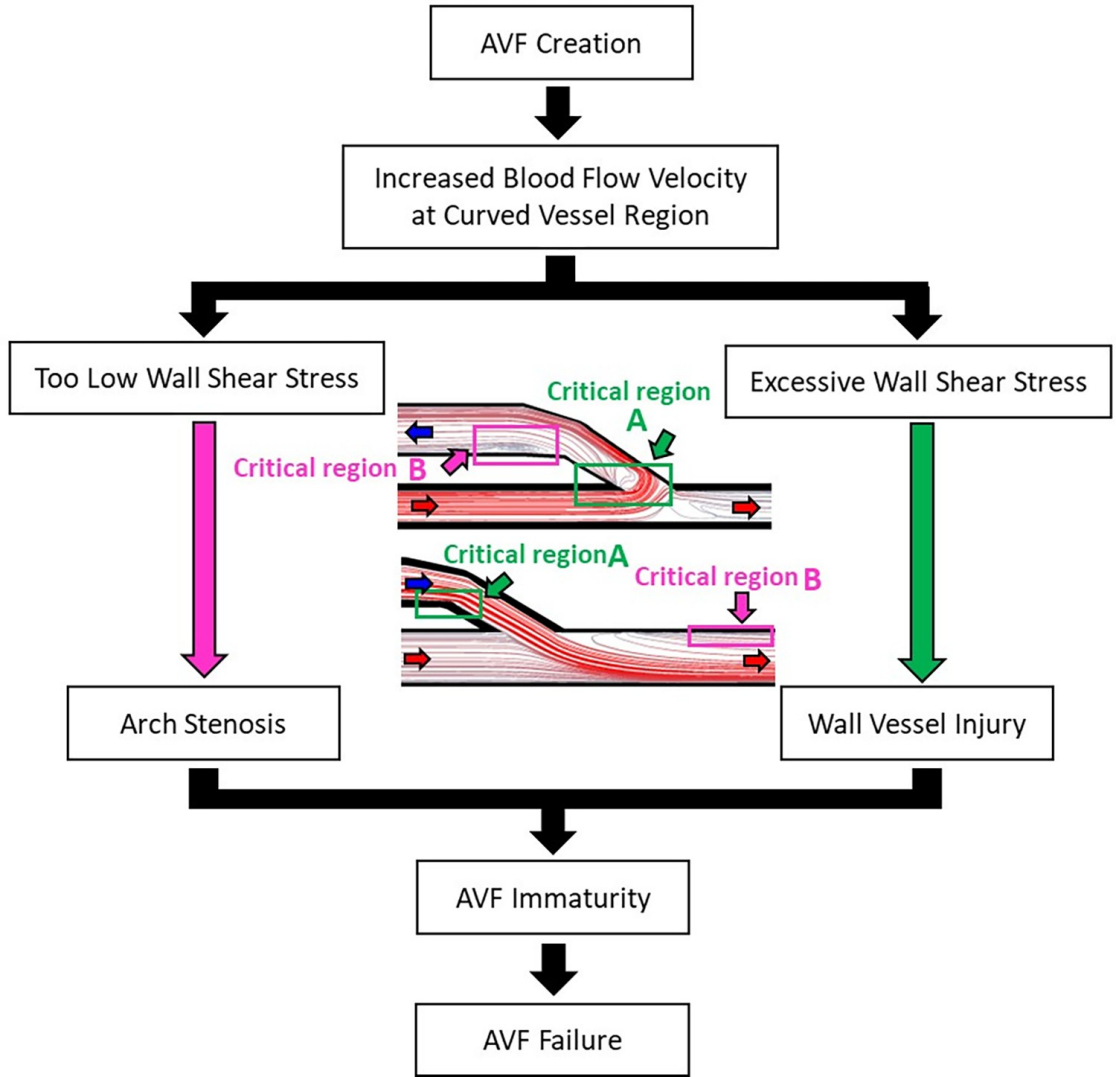
Diagrammatic of arteriovenous fistulas failure.

AVF creation results in high blood flow velocity that is diverted into the curved vessel, leading to abnormal hemodynamics in the region, including turbulent flow conditions and abnormal WSS [34–37]. Excessive WSS, ranging from 35–40 N/m^2^, can cause injury to the vessel wall within an hour of exposure [38]. Conversely, too low WSS, below 1 N/m^2^, has been shown to be associated with the development of stenosis formation from the WSS normalization process, which can contribute to AVF failure [6, 39–41]. Diagrammatic of AVF failure is demonstrated in Fig 1.

Furthermore, disturbed flow, characterized by low and reciprocating flow, may develop in zones of the AVF, indicating the sites of future stenoses [6]. Vessel stenosis formation tends to occur in areas of disturbed flow [32, 42, 43]. To reduce the rate of AVF immaturity from stenosis formation, hemodynamics should be optimized by creating more laminar flow transition at the curved vessel region as much as possible [44].

## Physical model

**Fig 2:**
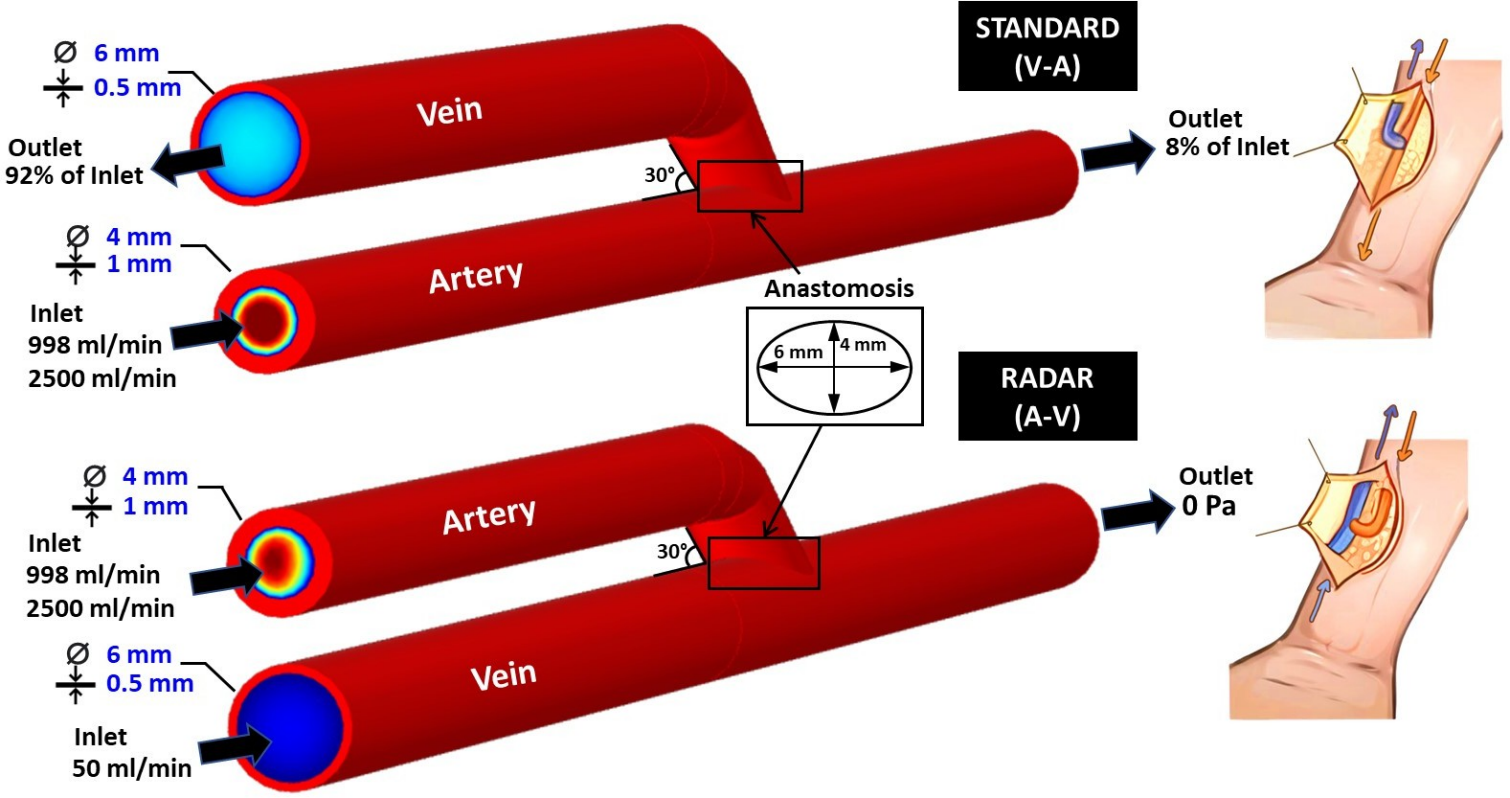
The schematic of the arteriovenous fistula models [45].

The schematic of the 3D AVF model, diameters, and AVF techniques; standard (V-A) and RADAR (A-V) approaches, are depicted in Fig 2. The numerical model was simulated using finite element analysis via COMSOL Multiphysics software. The mechanical properties of the blood, venous, and arterial vessels with different diseases used in the analysis, are provided in Table 1.

**Table 1:** Mechanical properties of blood vessel.

Parameter		Artery (Thickness 1 mm [46])		Vein (Thickness 0.5 mm [46])		Blood
Young’s modulus (kPa)	Healthy	100	[11]	25	[19]	-
	CKD	130				
	Diabetes			139.1		
	Hypertension			91.8		
Density (kg/m^3^)		1075 [47]		1056 [47]		1060 [47]
Poisson’s ratio (-)		0.46 [48]		0.48 [49]		-
Viscosity (Pa.s)		-		-		*μ*_*eff*_

## Methodology

In this study, fluid structure interaction is employed for analysis of 3D blood flow in elastic fistula. The blood is considered as fluid domain, and vessels (vein and artery) are considered as structural domain. The fluid-structure interfaces are expressed by acting forces between fluid and structure.

To simplify the problem, the following assumptions are made:

1. The blood flow inside AVF behaves as low-Reynolds turbulent flow.
2. The blood is assumed to be isotropic, homogeneous, non-Newtonian and incompressible fluid.
3. Deformations of fistula are small enough to be described by linear elasticity
4. The study is assumed to be time-independent.

This research undertaken herein was conducted without any involvement of human participants, ensuring adherence to ethical research practices. The medical data utilized in this study originates solely from peer-reviewed and established research within the relevant field.

### Blood flow analysis in fluid domain

The Wilcox low-Reynolds turbulence model [50–52] is utilized to simulate and predict turbulent arterial blood flow, as it is suitable for scenarios where laminar and turbulent flows coexist within certain areas of the arteries [52–54]. The set of equations that form the Wilcox low-Reynolds incompressible Navier-Stokes equation for turbulent flow include the continuity equation (Eq 1), which ensures mass conservation, the conservation of momentum in fluid flow (Eq 2), accounting for the forces acting on the fluid, the transport equation for turbulent kinetic energy, *k* (Eq 3), and the transport equation for the specific dissipation rate of turbulence, *ω* (Eq 4).

In *Ω*_*Fluid*_:

$$\frac{\partial u_{b}}{\partial x}+\frac{\partial v_{b}}{\partial y}+\frac{\partial w_{b}}{\partial z}=0$$ (1)

$$\rho_{b}\left(u_{b}\frac{\partial u_{b}}{\partial x}+v_{b}\frac{\partial u_{b}}{\partial y}+w_{b}\frac{\partial u_{b}}{\partial z}\right)=-\frac{\partial p}{\partial x}+\mu \left(\frac{\partial^{2}u_{b}}{\partial x^{2}}+\frac{\partial^{2}u_{b}}{\partial y^{2}}+\frac{\partial^{2}u_{b}}{\partial z^{2}}\right)$$

$$\rho_{b}\left(u_{b}\frac{\partial v_{b}}{\partial x}+v_{b}\frac{\partial v_{b}}{\partial y}+w_{b}\frac{\partial v_{b}}{\partial z}\right)=-\frac{\partial p}{\partial y}+\mu \left(\frac{\partial^{2}v_{b}}{\partial x^{2}}+\frac{\partial^{2}v_{b}}{\partial y^{2}}+\frac{\partial^{2}v_{b}}{\partial z^{2}}\right)$$ (2)

$$\rho_{b}\left(u_{b}\frac{\partial w_{b}}{\partial x}+v_{b}\frac{\partial w_{b}}{\partial y}+w_{b}\frac{\partial w_{b}}{\partial z}\right)=-\frac{\partial p}{\partial z}+\mu \left(\frac{\partial^{2}w_{b}}{\partial x^{2}}+\frac{\partial^{2}w_{b}}{\partial y^{2}}+\frac{\partial^{2}w_{b}}{\partial z^{2}}\right)$$

$$\left(\rho_{b}u_{b}\frac{\partial k}{\partial x}+\rho_{b}v_{b}\frac{\partial k}{\partial y}+\rho_{b}w_{b}\frac{\partial k}{\partial y}\right)=\frac{\partial }{\partial x}\left[(\mu +\frac{\mu_{t}}{\sigma_{k}})\frac{\partial k}{\partial x}\right]+\frac{\partial }{\partial y}\left[(\mu +\frac{\mu_{t}}{\sigma_{k}})\frac{\partial k}{\partial y}\right]+\frac{\partial }{\partial z}\left[(\mu +\frac{\mu_{t}}{\sigma_{k}})\frac{\partial k}{\partial z}\right]+G-\rho_{b}\omega k$$ (3)

$$\left(\rho_{b}u_{b}\frac{\partial \omega }{\partial x}+\rho_{b}v_{b}\frac{\partial \omega }{\partial y}+\rho_{b}w_{b}\frac{\partial \omega }{\partial y}\right)=\frac{\partial }{\partial x}\left[(\mu +\frac{\mu_{t}}{\sigma_{\omega }})\frac{\partial \omega }{\partial x}\right]+\frac{\partial }{\partial y}\left[(\mu +\frac{\mu_{t}}{\sigma_{\omega }})\frac{\partial \omega }{\partial y}\right]+\frac{\partial }{\partial z}\left[(\mu +\frac{\mu_{t}}{\sigma_{\omega }})\frac{\partial \omega }{\partial z}\right]+c_{1}\left(\frac{\omega }{k}\right)G-c_{2}\rho_{b}\omega^{2}$$ (4)

The turbulent viscosity is,

$$\mu_{t}=c_{\mu }\rho_{b}\frac{k}{\omega }$$ (5)

The generation rate of turbulence kinetic energy is as follows,

$$G=\mu_{t}\left(\frac{\partial u_{b}}{\partial x}+\frac{\partial v_{b}}{\partial y}+\frac{\partial w_{b}}{\partial z}\right)\left(\frac{\partial u_{b}}{\partial x}+\frac{\partial v_{b}}{\partial y}+\frac{\partial w_{b}}{\partial z}\right)$$ (6)

where *ρ*_*b*_ is the blood density (kg/m^3^), *u*_*b*_, *v*_*b*_, *w*_*b*_ represents the velocity component of blood in the direction of x, y, and z (m/s), *p* is the pressure (Pa), *k* is the turbulent kinetic energy (m^2^/s^2^), *ω* is the specific dissipation rate of turbulence (1/s), *μ* is the blood dynamic viscosity (Pa.s), *μ*_*t*_ is the turbulent viscosity (Pa.s), G is the generation of turbulence kinetic energy through mechanisms of velocity gradients (m^2^/s^3^).

The values of the Wilcox model constants are as follows [52],

*c*_1_ = 0.555, *c*_2_ = 0.8333, *c*_*μ*_ = 0.09, *σ*_*k*_ = 2, *σ*_*ω*_ = 2

The dynamic viscosity for non-Newtonian fluid in this study, is described by nonlinear function of shear rate of Carreau model [24–28, 54–56] as given;

$$\mu =\mu_{\infty }+(\mu_{0}-\mu_{\infty }){\left[1+{\left(\lambda \dot{\gamma }\right)}^{a}\right]}^{\frac{n-1}{a}}$$ (7)

where *μ*_0_ is denoted as zero shear rate viscosity (kg/m.s), *μ*_∞_ is the viscosity at the infinite shear rate (kg/m.s), *λ* is characteristic relaxation time, $\dot{\gamma }$ is scalar shear rate, *n* is flow index and *a* is dimensionless parameter describing the transition region between zero-shear-rate region and the power-law region. The Carreau model parameters for blood with different volume of red blood cells are indicated in Table 2. The non-linear values of shear-dependent blood viscosity of different blood characteristics are demonstrated.

**Table 2:** Carreau model parameters for blood at 37°C with different proportion of red blood cells.

the proportion of Red Blood Cells	*μ*_0_ (kg/m.s)	*λ*	*n*	*μ*_∞_ (kg/m.s)	Reference
45% (Normal)	0.056	3.313	0.356	0.00345	[54, 57, 58]
15%	0.0273	3.314	0.354	0.001	[58]
5%	0.0233	3.313	0.352	0.002	[58]

#### Boundary conditions

The vessel walls are held under non-slip conditions. At the entrance of the fistula, laminar flow is assumed with very low values of turbulence parameters, represented by the *k*−*ω* model, where *k* is set to 0.0001 and *ω* to 0.45 [52]. Meanwhile, at the outlet, fully developed flow is considered.

For standard technique (V-A), the inflow at the arterial inlet is applied with 998 ml/min [59–65] under normal circumstance, 2500 ml/min [66–69] for excessive high flow (hypertension). The outflows are applied with a flow distribution of 8% and 92% towards the arterial outlet and venous outlet, respectively.

For RADAR technique (A-V), the inflow at arterial inlet is applied as the same amount as standard technique. The inflow at venous inlet is applied with 50 ml/min [70] and flow out at venous outlet with pressure 0 Pa. The flow rates in this study are referred to the value of radiocephalic AVF.

This study has a limitation where the outlet pressure is assumed to be 0 Pa, simplifying the model. While this assumption might not notably influence the overall result of WSS, it could affect the dynamics of blood flow within the fistula. Therefore, considering precise values, such as an outlet pressure of 5 mmHg, can significantly enhance the accuracy of predictions. Hence, it is recommended that future studies utilize more precise boundary conditions to better represent real-world scenarios.

### Mechanical deformation analysis in structural domain

The displacement of structure in for vessel domain is described in this section. The equations of momentum in the structural domain are as follow [71];

In *Ω*_*Solid*_:

$$\rho_{v}u_{v}=\frac{\partial \sigma_{xx}}{\partial x}+\frac{\partial \tau_{xy}}{\partial y}+\frac{\partial \tau_{xz}}{\partial z}+{\rho_{v}f}_{F}$$

$$\rho_{v}v_{v}=\frac{\partial \tau_{xy}}{\partial x}+\frac{\partial \sigma_{yy}}{\partial y}+\frac{\partial \tau_{yz}}{\partial z}+{\rho_{v}f}_{F}$$ (8)

$$\rho_{v}w_{v}=\frac{\partial \tau_{xz}}{\partial x}+\frac{\partial \tau_{yz}}{\partial y}+\frac{\partial \sigma_{zz}}{\partial z}{+\rho_{v}f}_{F}$$

Where *u*_*v*_, *v*_*v*_, *w*_*v*_ represent the displacement of vessel(mm), *ρ*_*v*_ is vessel density (kg/m^3^), *σ* is the stress (N/m^2^), *τ* is wall shear stress (N/m^2^), and *f*_*F*_ is applied body force from fluid (N). The equilibrium equations for solid mechanics, written in a Cartesian coordinate system, are [72, 73]:

$$\frac{\partial \sigma_{xx}}{\partial x}+\frac{\partial \tau_{xy}}{\partial y}+\frac{\partial \tau_{xz}}{\partial z}=0$$

$$\frac{\partial \tau_{xy}}{\partial x}+\frac{\partial \sigma_{yy}}{\partial y}+\frac{\partial \tau_{yz}}{\partial z}=0$$ (9)

$$\frac{\partial \tau_{xz}}{\partial x}+\frac{\partial \tau_{yz}}{\partial y}+\frac{\partial \sigma_{zz}}{\partial z}=0$$

The stress–strain relationship (Eq (10)) and the strain-displacement relationship (Eq (11)) are as follows [72, 73]:

$$\varepsilon_{xx}=\frac{1}{E}[\sigma_{xx}-\nu (\sigma_{yy}+\sigma_{zz})]$$

$$\varepsilon_{yy}=\frac{1}{E}[\sigma_{yy}-\nu (\sigma_{xx}+\sigma_{zz})]$$

$$\varepsilon_{zz}=\frac{1}{E}[\sigma_{zz}-\nu (\sigma_{xx}+\sigma_{yy})]$$ (10)

$$\varepsilon_{xy}=\sigma_{xy}\left(1+\nu \right)/E$$

$$\varepsilon_{xz}=\sigma_{xz}\left(1+\nu \right)/E$$

$$\varepsilon_{yz}=\sigma_{yz}\left(1+\nu \right)/E$$

$$\varepsilon_{xx}=\frac{\partial u_{v}}{\partial x},\varepsilon_{yy}=\frac{\partial v_{v}}{\partial y},\varepsilon_{zz}=\frac{\partial w_{v}}{\partial z}$$

$$\varepsilon_{xy}=\frac{1}{2}\left(\frac{\partial u_{v}}{\partial y}+\frac{\partial v_{v}}{\partial z}\right)$$ (11)

$$\varepsilon_{xz}=\frac{1}{2}\left(\frac{\partial u_{v}}{\partial z}+\frac{\partial w_{v}}{\partial x}\right)$$

$$\varepsilon =\frac{1}{2}\left(\frac{\partial v_{v}}{\partial z}+\frac{\partial w_{v}}{\partial y}\right)$$

where *ε* denotes the mechanical strain, *E* is Young’s modulus (Pa), *ν* is the Poisson’s ratio. The mechanical strain is calculated by Young’s modulus (Pa) and the Poisson’s ratio, where demonstrate in Table 1.

#### Boundary conditions

To predict future stenosis in a fistula, a crucial consideration is the primary generation of WSS specifically occurring at the anastomosis area. The dynamic interaction between blood flow and the vessel wall within the fistula significantly influences the WSS, potentially leading to future stenosis. Consequently, the boundary conditions applied to the surfaces of the anastomosis angle are defined as free boundary conditions, allowing movement. Meanwhile, the remaining surfaces are designated as fixed boundary conditions, effectively constraining any movement of the structure, and thereby simulating the behavior of surrounding tissues.

### Fluid-structure interaction

To satisfy the blood flow in elastic vessel analysis, the deformable moving mesh is used. The fluid structural interaction is employed at the blood-vessel interface as follows [71, 74],

$$\Gamma_{fs}=\Omega_{f}+\Omega_{s}$$ (12)

Fluid solver of displacement from solid,

$$\Omega_{f}:f_{F}=F(d_{S})$$ (13)

Solid solver of force from fluid,

$$\Omega_{s}:d_{s}=S(f_{F})$$ (14)

The conditions on the fluid-solid interior boundary can be described as follow,

$${\left[u_{b},v_{b},w_{b}\right]}_{f}={\left[u_{v},v_{v},w_{v}\right]}_{s}\;at\;\Gamma_{fs}$$

$$\sigma_{s}={-\sigma }_{f}\;at\;\Gamma_{fs}$$

### Verification of the model

**Fig 3:**
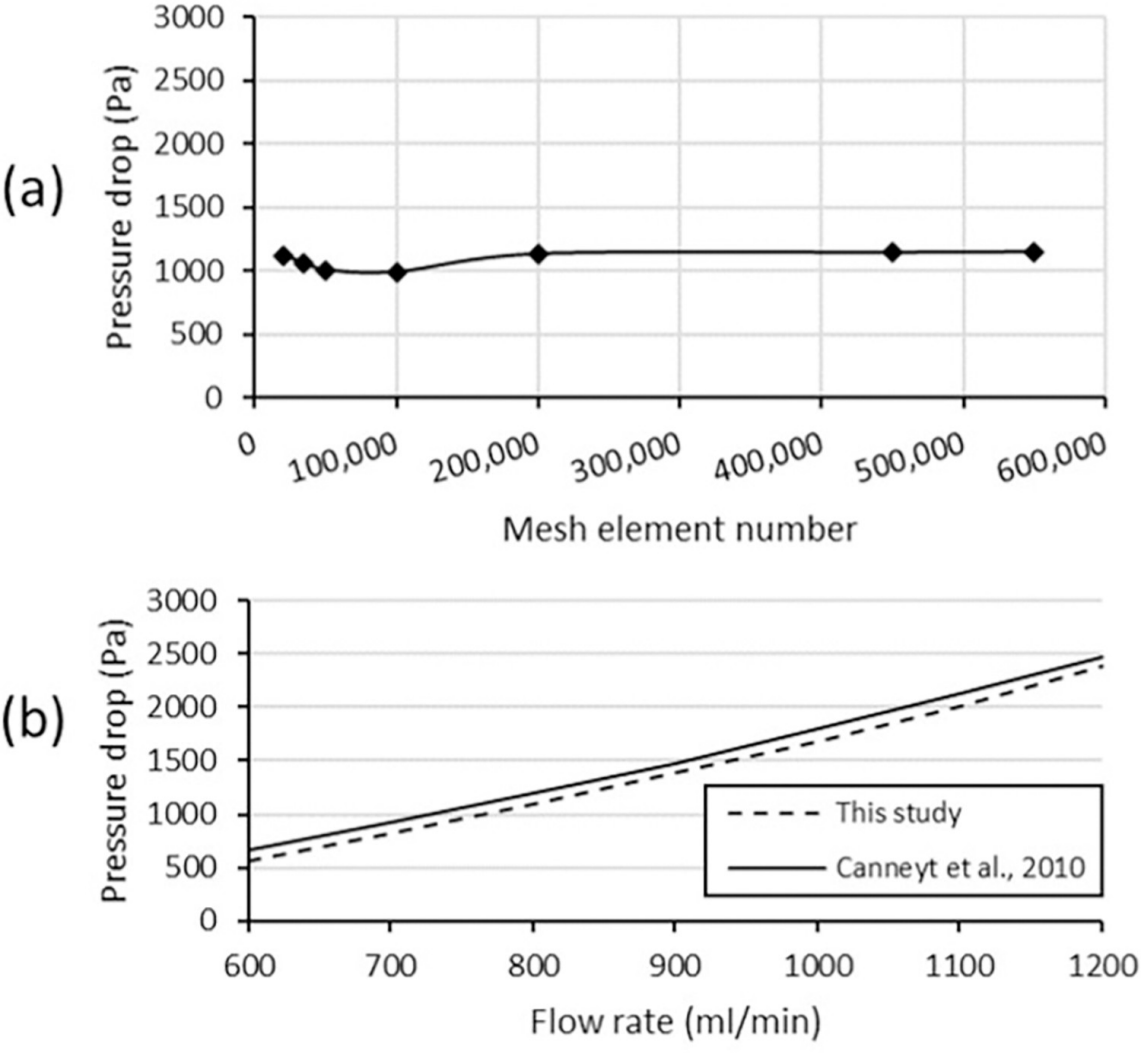
(a) Mesh convergence curve of the model (b) Validation result compared between this study and Canneyt et al., 2010 [45].

This model was simulated using a reasonable number of elements, as shown in Fig 3(A). A mesh element count of 600,000 was employed in this study. Furthermore, the accuracy of our model was verified by comparing the simulation results with relevant literature [45]. Specifically, pressure drops were compared between the simulation data and experimental data, as depicted in Fig 3(B). The results clearly demonstrate the accuracy of our model, as evidenced by the close match between the findings of this study and the previous work.

## Results

AVF Thrombus is a significant risk factor for patients with CKD, and it is often associated with hypertension, anemia, and diabetes [75]. These diseases can impact blood vessel elasticity and affect blood flow patterns and WSS. In this study, two techniques, standard and improved RADAR, are compared. The simulation of WSS helps predict potential damage to the vessel wall at critical region A (WSS > 35 N/m^2^) and the formation of stenosis at critical region B (WSS < 1 N/m^2^).

### Effect of vessel elasticity

#### Effect of diabetes disease on artery and vein stiffness

**Fig 4:**
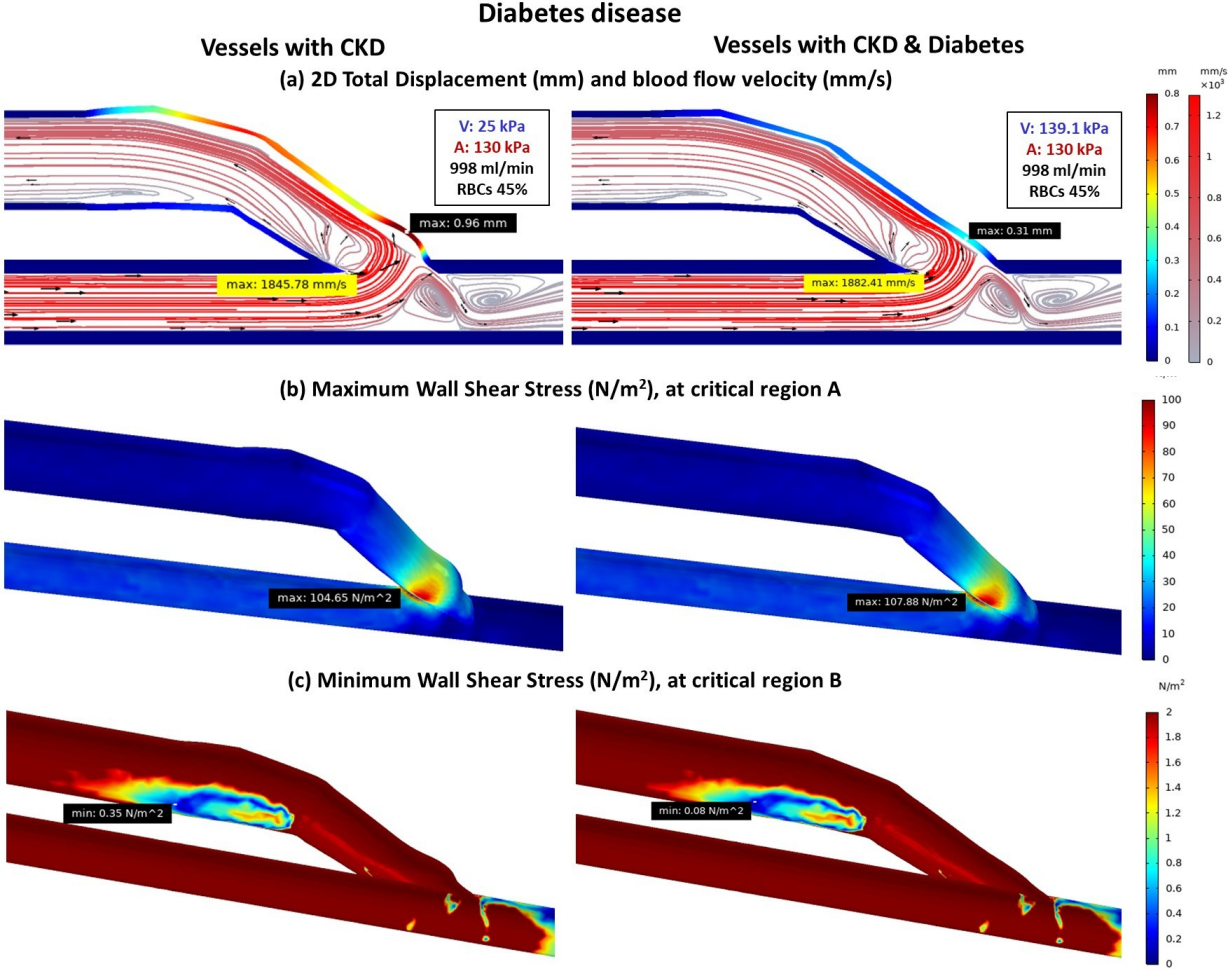
The comparison of fistula with CKD and fistula with CKD & diabetes disease (a) Total displacement of vessels and blood flow velocity (b) Maximum wall shear stress at critical region A (c) Minimum wall shear stress at critical region B.

**Fig 5:**
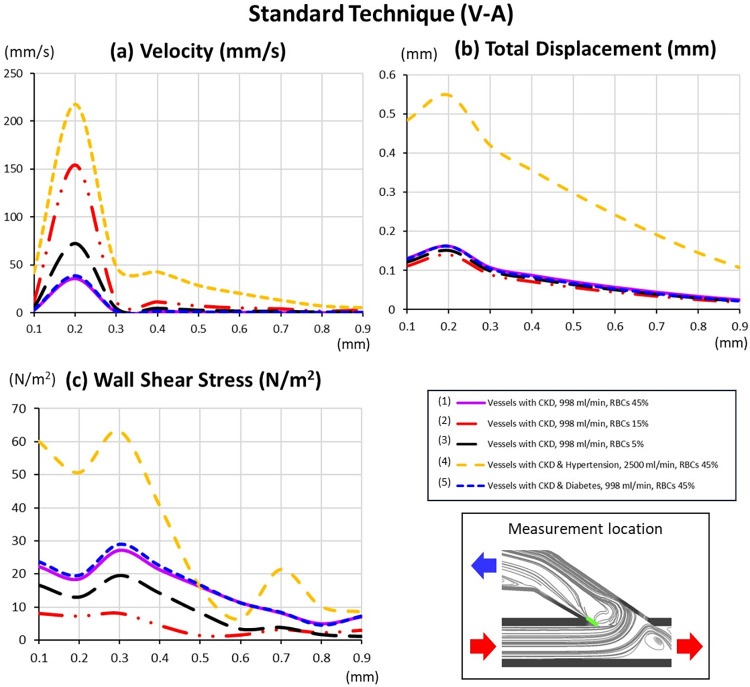
Standard technique (V-A); Blood flow velocity, vessel total displacement and WSS between of fistula with different diseases.

The combination of CKD and diabetes causes increased stiffness in both artery and vein [11–14] that leads to changes in blood flow patterns and interactions with the vessel wall (Fig 4(A)). In standard AVF model, veins with higher stiffness experience less deformation and slower velocity at the anastomosis joint connection that result in higher WSS at critical region A (Figs 4(B) and 5; lines 1 and 5). However, the flow becomes faster after the curvature of anastomosis that leading to lower WSS at critical region B (Fig 4(C)).

#### Effect of hypertension on blood flow rate and artery stiffness

**Fig 6:**
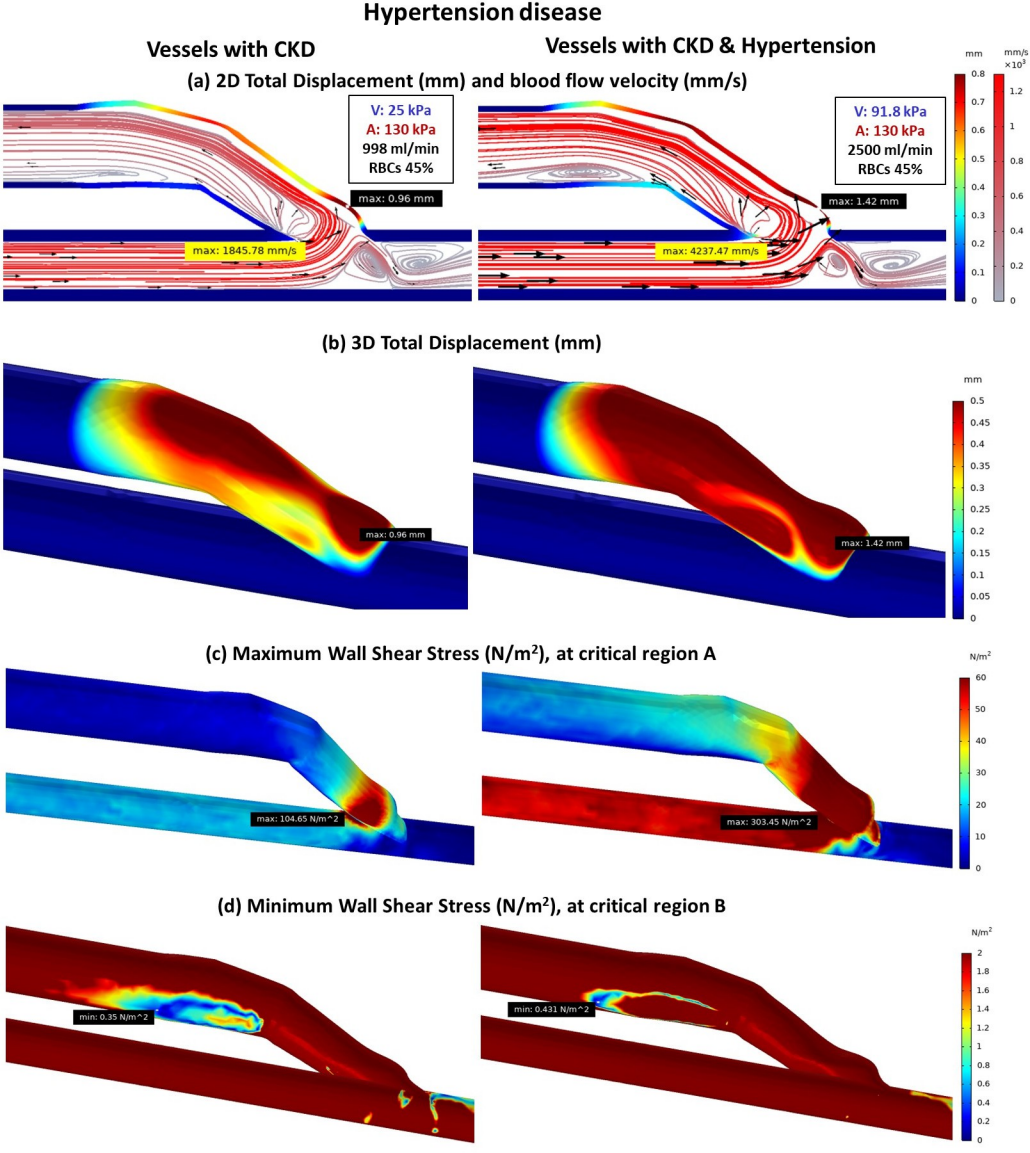
The comparison of fistula with CKD and fistula with CKD & hypertension disease (a) 2D total displacement of vessels and blood flow velocity (b) 3D total displacement of vessels (c) Maximum wall shear stress at critical region A (d) Minimum wall shear stress at critical region B.

Hypertension leads to excessive blood flow rate (2500 ml/min) and increased stiffness in both artery and vein. In standard AVF model, the additional flow provides a higher attacking force on the less deformed vessel wall at the anastomosis area resulting in potentiation of WSS elevation over that from increased stiffness alone. This can potentially cause injury to the inner vessel wall, especially in critical region A (Fig 5(B) and 5(C); lines 1 and 4, and Fig 6(B) and 6(C)). However, the generated turbulent flow pattern at critical region B due to faster flow (Fig 6(A)) is shown to be more favorable than that in CKD without hypertension. It results in a smaller area of insufficient WSS at critical region B, as faster flow can increase WSS generation (Fig 6(D)).

### Effect of blood property

#### Effect of anemia disease on blood viscosity

**Fig 7:**
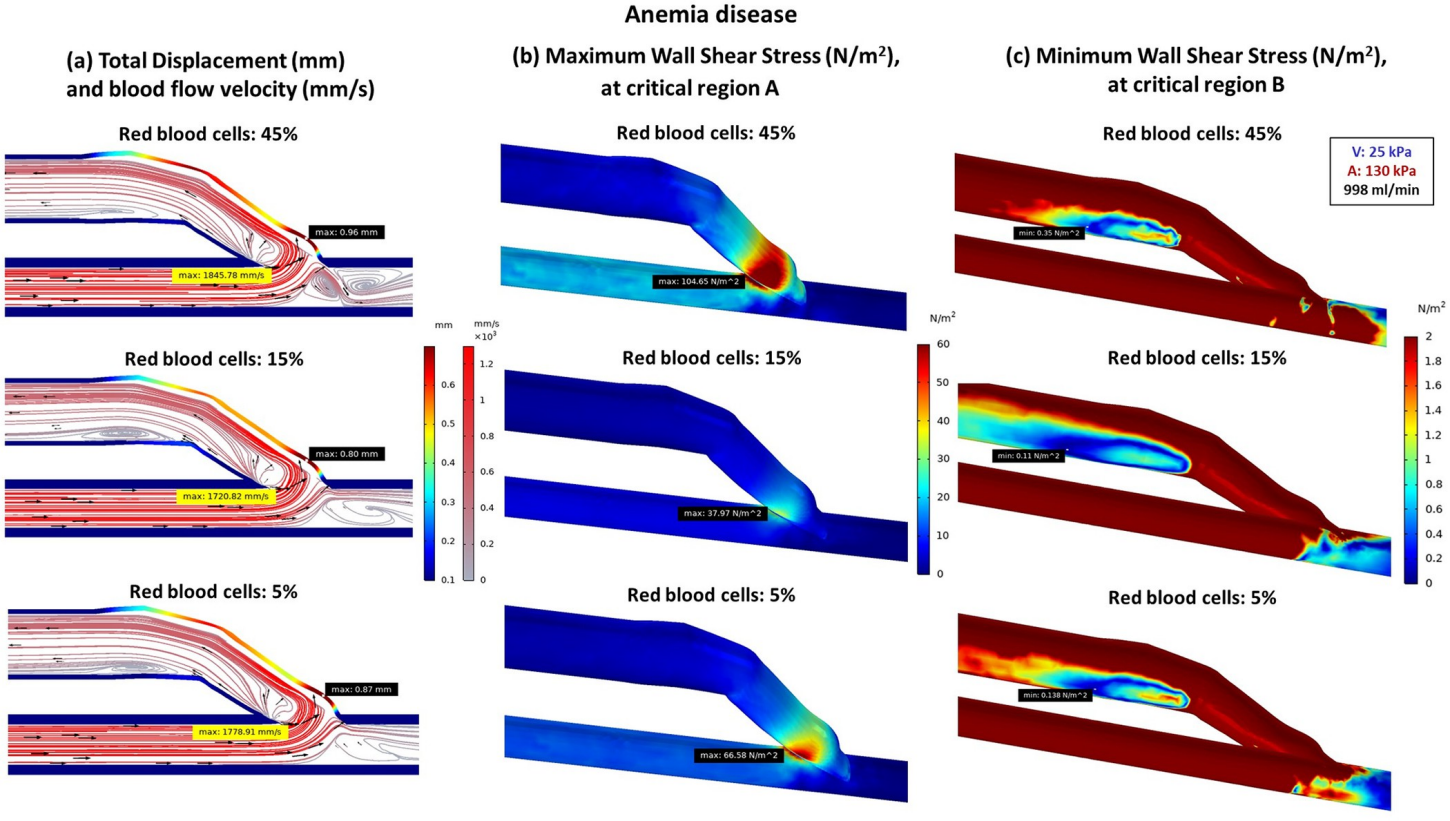
The comparison of total displacement, blood flow velocity and WSS at critical regions, between different red blood cells proportion of (a) 45% (b) 15% (c) 5%.

Anemia is a condition that occurs when a patient has a lower number of RBCs. This characteristic of blood is associated with the non-Newtonian fluid property, where viscosity varies depending on the proportion of RBCs (Table 2). The RBC levels of 45%, 15%, and 5% are considered normal [54, 57, 58], lower than usual [58], and insufficient RBCs conditions [58], respectively. When the RBC level is at 45%, the blood tends to have the highest viscosity, resulting in slower blood flow velocity (Fig 5(A); lines 1–3). Consequently, it encounters higher resistance within the blood vessels, leading to greater vessel deformation and higher WSS (Fig 5(B) and 5(C); lines 1–3, and Fig 7(A)–7(C)). In the standard AVF model, the most disturbed blood flow, fastest velocity, and lowest WSS are observed in red blood cells at 15% (Fig 5; lines 1–3 and Fig 7(A)–7(C)).

### Effect of surgical technique

#### Comparison between standard AVF and RADAR techniques

**Fig 8:**
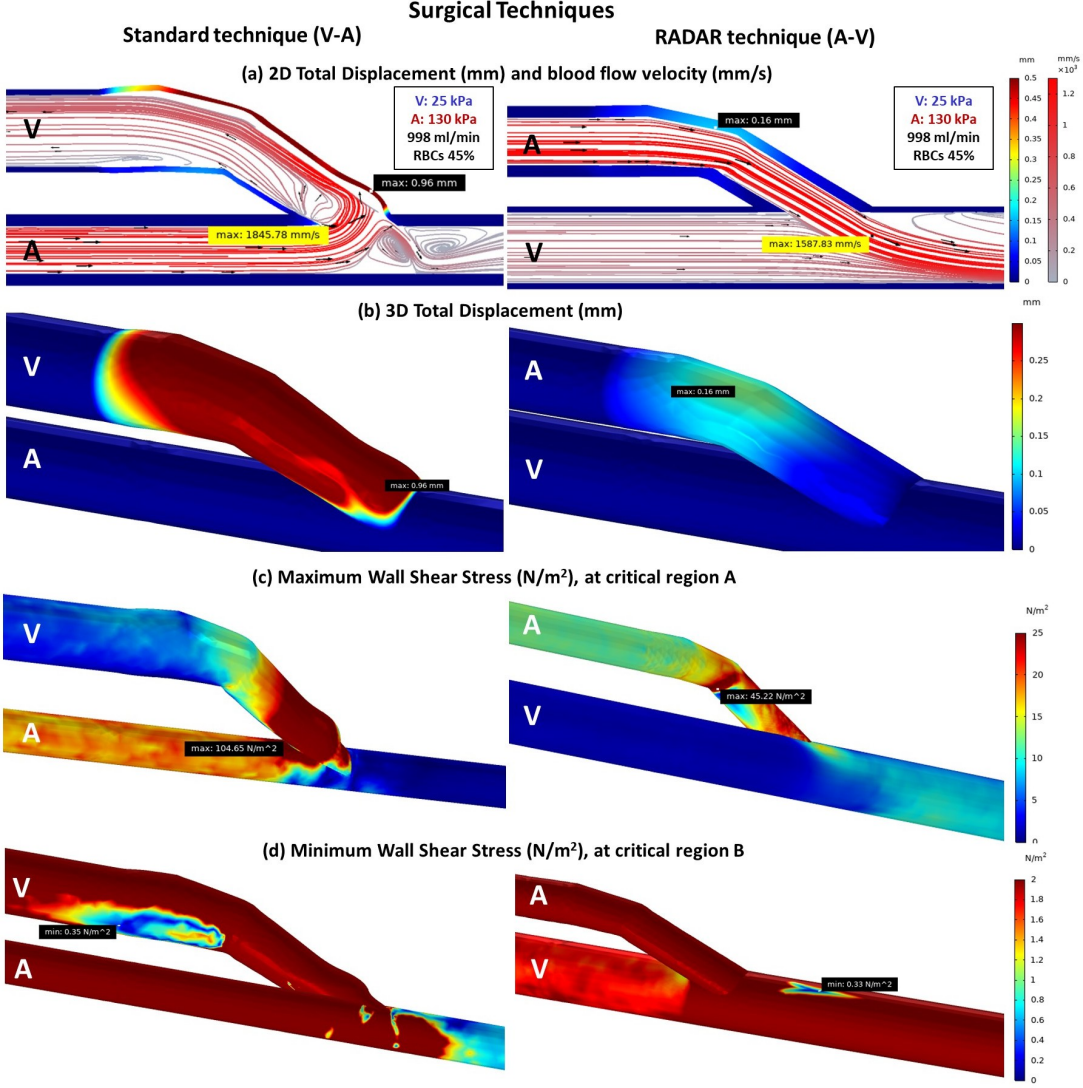
The comparison of standard and RADAR techniques of fistula with CKD (a) 2D total displacement of vessels and blood flow velocity (b) 3D total displacement of vessels (c) Maximum WSS at critical region A (d) Minimum WSS at critical region B.

The RADAR technique generates a smoother flow along the anastomosis and produces less turbulence within the fistula compared to the standard technique (Fig 8(A)). This smoother flow results in a lower velocity and reduces the force attacking the vessel wall, resulting in less deformation (Fig 8(B)). As a consequence, the RADAR technique leads to significantly lower WSS levels at critical region A and slightly lower WSS at critical region B (Fig 8(C) and 8(D)). Notably, the highest WSS is generated in the area of blood inflow to the anastomosis for both techniques, which is considered as critical region A.

#### Effect of hypertension, and anemia diseases on vessels stiffness and blood flow phenomena

**Fig 9:**
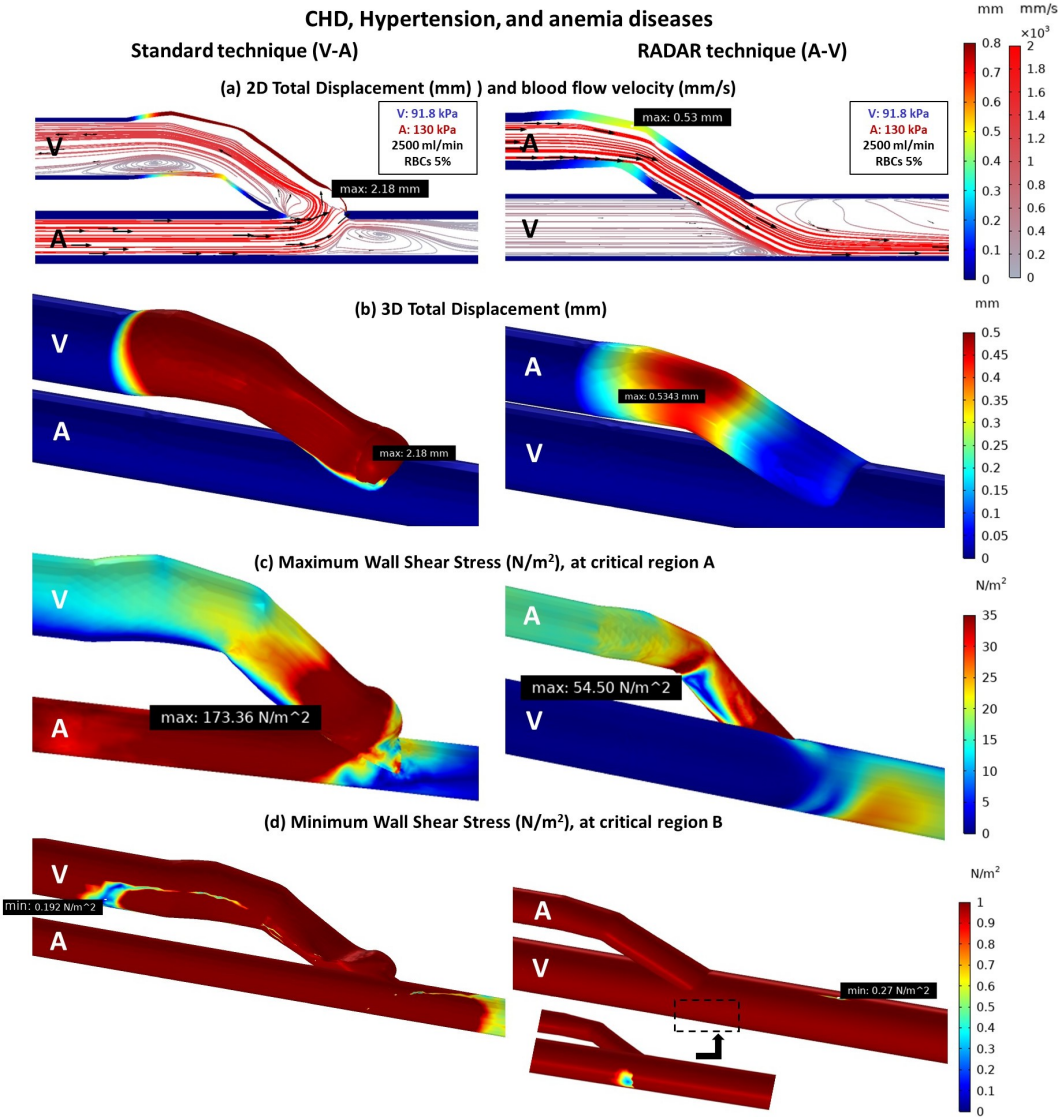
The comparison of standard and RADAR techniques of fistula with CKD, hypertension, and anemia diseases (a) 2D total displacement of vessels and blood flow velocity (b) 3D total displacement of vessels (c) Maximum WSS at critical region A (d) Minimum WSS at critical region B.

In high-risk cases of AVF maturity failure, such as hypertension and anemia, where the veins and arteries become stiffer, blood viscosity significantly decreases, and blood flow rate increases (2500 ml/min). The results demonstrate that the RADAR technique generates a more favorable flow compared to the standard technique. Only a small turbulence is created under the main flow from the artery (Fig 9(A)). The anastomosis area, which is most affected by the blood flow, experiences less deformation in the RADAR technique (Fig 9(B)), resulting in a significantly smaller WSS at critical region A (Fig 9(C)). However, the presence of small turbulence can lead to a small area of insufficient WSS beneath the flow at the anastomosis (Fig 9(D)).

#### Effect of hypertension, anemia and diabetes diseases on vessels stiffness and blood flow phenomena

**Fig 10:**
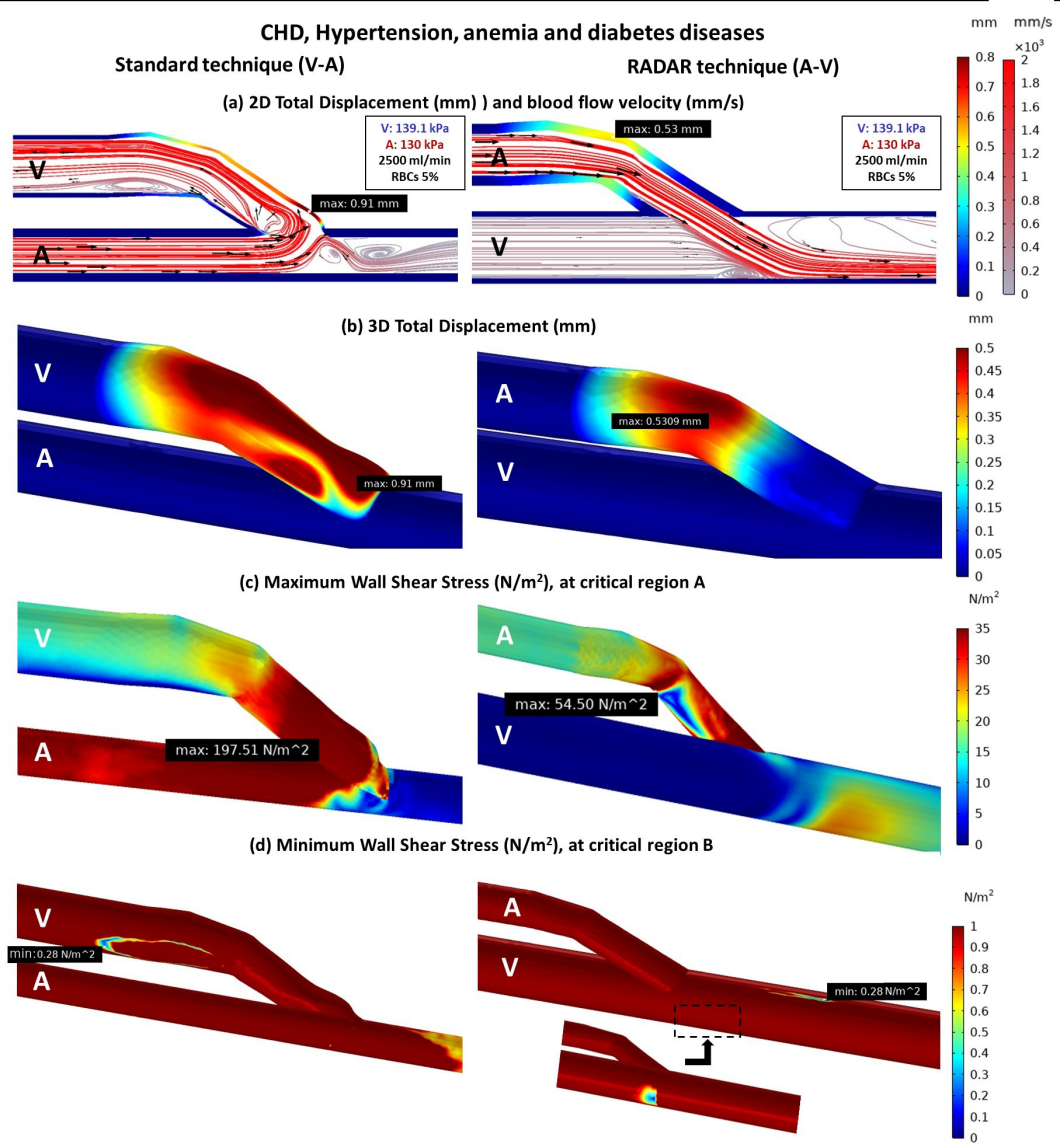
The comparison of standard and RADAR techniques of fistula with CKD, hypertension, anemia, and diabetes diseases (a) 2D total displacement of vessels and blood flow velocity (b) 3D total displacement of vessels (c) Maximum WSS at critical region A (d) Minimum WSS at critical region B.

Diabetes is often associated with CKD, hypertension, and anemia, leading to increased stiffness in venous vessels. In the standard technique, where the anastomosis is made by vein, there is less deformation compared to the case without diabetes (Figs 9(B) and 10(B)). This results in an altered blood flow pattern (Figs 9(A) and 10(A)) and higher WSS at critical region A, which is more noticeable in the standard technique due to the vein is role as the anastomosis part in this technique (Figs 9(C) and 10(C)).

**Fig 11:**
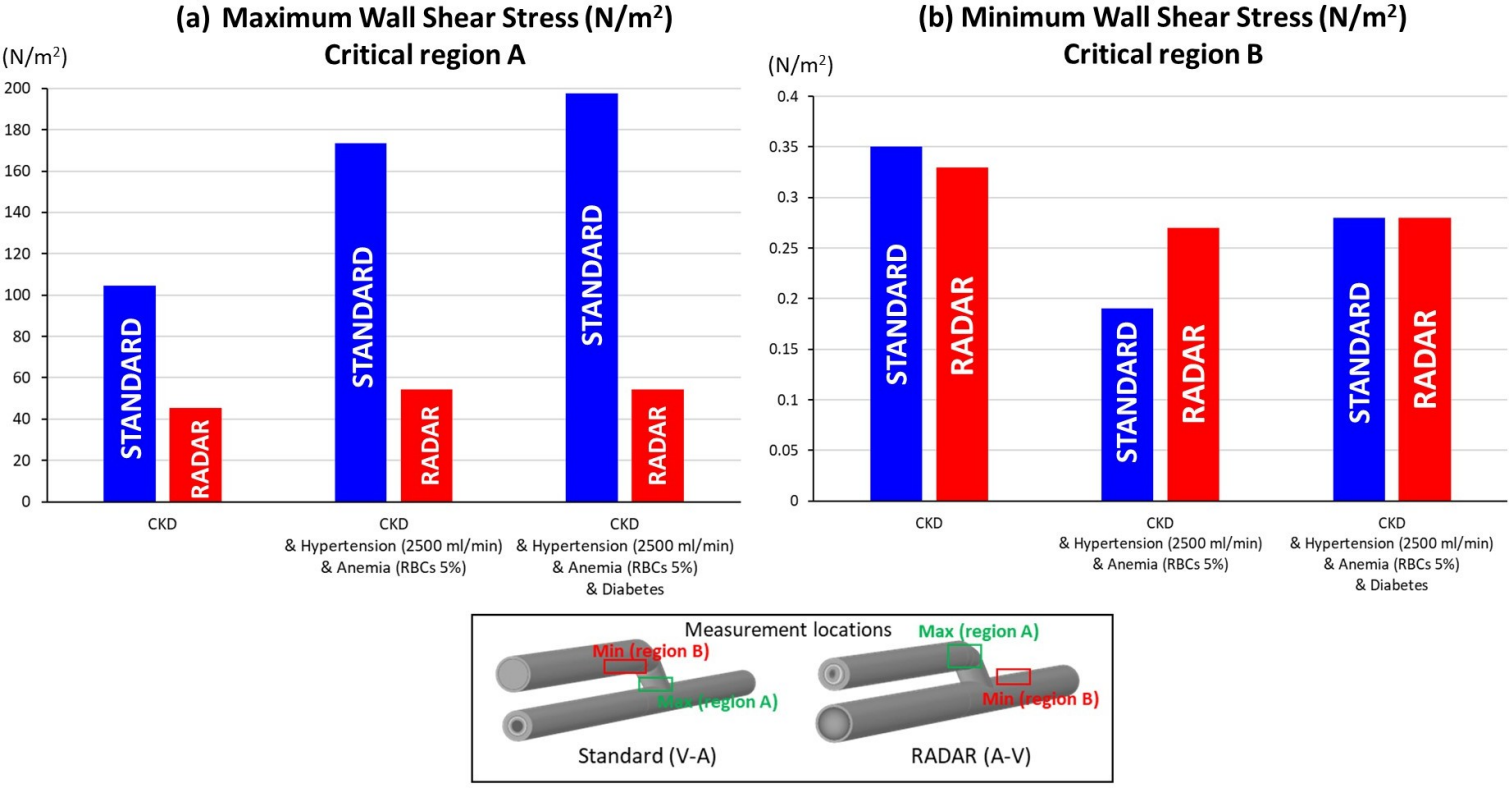
The comparison between standard and RADAR techniques with/without sets of diseases (a) Maximum WSS at critical region A (b) Minimum WSS at critical region B.

When comparing the cases with and without additional diseases using the two surgical techniques, the RADAR technique consistently generates less WSS at critical region A (Figs 8(D), 9(D) and 10(D)). However, the WSS value at critical region B remains approximately the same for both techniques (Fig 11(A) and 11(B)).

## Discussion

### Diabetes and hypertension diseases

The presence of vessel elasticity plays a crucial role in assessing the risk of complications related to maturity failure in patients with diabetes and hypertension. In diabetes, changes in vessel stiffness can lead to altered blood flow patterns and varying levels of WSS, which contribute to a slightly higher chance of stenosis development. In patients with hypertension, the effects of vessel stiffness are further amplified by the excessive blood flow rate. This combination results in excessively unfavorable WSS at the anastomosis, causing a significant damage to the vessel walls and posing a greater risk of complications such as fistula immaturity. However, the likelihood of stenosis formation from insufficient WSS is relatively lower compared to cases without hypertension.

### Anemia disease

Accelerated blood flow exerts a stronger force on vessel walls, causing increased impact and leading to the emergence of turbulent flow through enhanced blood reflection. In cases of anemia, characterized by lower red blood cell counts, blood becomes less viscous, contributing to faster blood velocity and a decrease in WSS generation. This occurrence potentially reduce damage to vessel walls, especially at critical area A, where minimizing WSS is crucial for maintaining vascular health. However, a complication arises at critical area B due to turbulent flow, resulting in insufficient WSS, where maximizing WSS is necessary to prevent potential future stenosis resulting from its deficiency. Consequently, a lower number of RBCs may increase the possibility of stenosis formation in the future.

In a non-Newtonian fluid like blood, the flow behavior is influenced by multiple factors, including the concentration of RBCs, which adds complexity to the actual blood flow characteristics. The interaction of these factors can give rise to unexpected velocity patterns, where the blood velocity may not strictly follow the order of RBC levels. Previous studies have also noted this non-linear relationship between RBC concentration and blood flow velocity [58, 76]. Additionally, factors such as temperature may contribute to RBCs aggregate size and blood viscosity [58]. Therefore, these factors collectively explain the occurrence of unexpected flow phenomena observed when RBCs are present at a concentration of 15%.

In clinical scenarios, sub-normal maintenance in anemia treatment would be crucial to provide a favorable WSS to critical region A to minimize risk of vessel injury. However, too low concentration of RBCs may negatively affect the critical region B as previously mentioned. Furthermore, there is currently a lack of comprehensive information regarding the behavior of blood flow related to anemia, particularly concerning the viscosity of blood flow using a non-Newtonian model, which exhibits a non-linear pattern. Therefore, conducting additional studies is essential to enhance our understanding of how anemia-related non-Newtonian blood flow influences WSS in AVF.

### Standard and RADAR techniques

The RADAR technique outperforms the standard technique in multiple aspects. It increases WSS at critical region B, where insufficient WSS poses a risk, while reducing WSS at critical region A to minimize the risk of wall damage. Moreover, due to its thicker vessel, the RADAR technique lowers the chance of vessel injury compared to the standard technique. However, it is essential to consider that the smaller area of insufficient WSS (critical region B) in RADAR may still present a risk of future stenosis formation.

When considering the combination of CKD, hypertension, and anemia, the RADAR technique proves to be superior in terms of blood flow and WSS generation. It facilitates a smoother flow and lowers WSS at critical region A, which is essential in reducing the risk of complications. Notably, even in the presence of diabetes, the RADAR technique maintains its superior performance and remains unaffected by the stiffer veins associated with diabetes. On the other hand, the standard technique yields poorer results, as the use of the vein for anastomosis can be negatively impacted by the presence of stiffer veins in diabetic patients. The RADAR technique proves to be more effective in managing the complexities of CKD, hypertension, and anemia, while also remaining resilient in the presence of diabetes-related stiffer veins. This makes RADAR a favorable choice for optimizing blood flow and minimizing potential complications.

Regarding the possibility of stenosis formation in cases with hypertension and anemia, the RADAR technique shows two small areas where the risk of stenosis may be higher over time. It is possible that this risk can be mitigated by adjusting the anastomosis angle to be smaller than 30° for smoother flow and better WSS generation, but further studies are required to confirm this prospect. Although RADAR technique possesses a positive change in hemodynamic for AVF maturation, the risk of sacrificing the distal flow should be taken into the account to minimize other complications related to the decreased blood flow.

## Conclusion

The physiologic consequences of diabetes and hypertension pose a significant risk for AVF maturation failure. In contrast, the anemic condition resulting from CKD may help reduce vessel injury but raises concerns about potential stenosis formation. However, due to the complexity of viscosity-dependent blood behavior associated with anemia, further studies are needed to better understand its influence on AVF maturation. Additionally, diseases related to blood properties, such as low-density lipoprotein, could significantly affect surgical outcomes [77] and should be considered in future research. Despite the presence of these co-morbidities, the RADAR technique has demonstrated its efficacy in providing more favorable hemodynamic alterations for AVF maturation.
